# Screening and evaluation of drought resistance traits of winter wheat in the North China Plain

**DOI:** 10.3389/fpls.2023.1194759

**Published:** 2023-06-16

**Authors:** Xiaoyuan Bao, Xiaoyang Hou, Weiwei Duan, Baozhong Yin, Jianhong Ren, Yandong Wang, Xuejing Liu, Limin Gu, Wenchao Zhen

**Affiliations:** ^1^ College of Agronomy, Hebei Agricultural University, Baoding, China; ^2^ Key Laboratory of North China Water-saving Agriculture, Ministry of Agriculture and Rural Affairs, Baoding, China; ^3^ State Key Laboratory of North China Crop Improvement and Regulation, Hebei Agricultural University, Baoding, China; ^4^ College of Clinical Medicine, North China University of Science and Technology, Tangshan, China

**Keywords:** winter wheat, drought resistance, screening, principal component analysis, comprehensive evaluation

## Abstract

**Background:**

Drought-resistant varieties are an important way to address the conflict between wheat’s high water demand and the scarcity of water resources in the North China Plain (NCP). Drought stress impacts many morphological and physiological indicators in winter wheat. To increase the effectiveness of breeding drought-tolerant varieties, choosing indices that can accurately indicate a variety’s drought resistance is advantageous.

**Results:**

From 2019 to 2021, 16 representative winter wheat cultivars were cultivated in the field, and 24 traits, including morphological, photosynthetic, physiological, canopy, and yield component traits, were measured to evaluate the drought tolerance of the cultivars. Principal component analysis (PCA) was used to transform 24 conventional traits into 7 independent, comprehensive indices, and 10 drought tolerance indicators were screened out by regression analysis. The 10 drought tolerance indicators were plant height (PH), spike number (SN), spikelet per spike(SP), canopy temperature (CT), leaf water content (LWC), photosynthetic rate (A), intercellular CO2 concentration (Ci), peroxidase activity (POD), malondialdehyde content (MDA), and abscisic acid (ABA). In addition, through membership function and cluster analysis, 16 wheat varieties were divided into 3 categories: drought-resistant, drought weak sensitive, and drought-sensitive.

**Conclusion:**

JM418, HM19,SM22, H4399, HG35, and GY2018 exhibited excellent drought tolerance and,therefore, can be used as ideal references to study the drought tolerance mechanism in wheat and breeding drought-tolerant wheat cultivars.

## 1 Introduction

Drought is an abiotic stress with extensive crop yield losses and has become an important constraining factor of agricultural development (Fang and Xiong, 2015). As one of the driest regions in the world, the North China Plain (NCP) is China’s most important wheat-producing region, accounting for about 71% of the country’s total wheat production (Sun et al., 2006). The region has a temperate monsoon climate, with 50–150 mm of precipitation falling during the wheat-growing season (Fang et al., 2010; Jha et al., 2017). Therefore, the spring drought has become the predominant drought pattern affecting the wheat-growing period (Wu et al., 2021). To increase grain production, farmers in the NCP tapped groundwater for irrigation, causing water table declines and producing the largest funnel area in the world, with serious hydrological consequences (Liu et al., 2022).

Many strategies were implemented to alleviate the conflict between wheat production and water scarcity, including the planting of drought-resistant wheat varieties, the use of water-saving irrigation equipment, and water-saving irrigation field management (Morison et al., 2008). Among these, growing drought-resistant wheat varieties is the foundation for increased yield and water productivity. Efforts to reduce the impact of drought by breeding drought-resistant cultivars have been underway worldwide for a long time, but their progress is influenced by the environment (Mwadzingeni et al., 2016). Meanwhile, drought resistance is a complex trait controlled by a large number of genes, resulting from the interaction between different basic constituents or adaptive traits, each of which may be subject to complex genetic and environmental changes. Therefore, developing and evaluating drought-tolerant crop varieties and screening for drought-tolerant traits are necessary to ensure sustainable food production in future climate scenarios (Gambetta et al., 2020).

The morphological and physiological responses of crops to drought can explain the large variation in yield under drought stress conditions (Puangbut et al., 2022). From a morphological perspective, drought reduced the size of wheat organs, such as leaves, stems, ears, and tillers, at different developmental stages (Ihsan et al., 2016). Therefore, plant height, panicle number, spikelet seed setting rate, 1000-grain weight, and aboveground biomass all decreased (Li et al., 2010; Gao et al., 2020; Memon et al., 2021). At the canopy level, drought resulted in increased canopy temperature and decreased leaf area index. In addition to the phenotypic changes caused by drought stress, many physiological and biochemical changes occur in crops to withstand the negative effects of adversity. Photosynthesis is the basis of plant growth and the primary metabolic process in plants, and it can be disrupted by stress even at low levels (Fan et al., 2019). Carbon uptake during photosynthesis is intimately connected with wheat productivity and is also crucially controlled by stomatal opening (Correia et al., 2022). Plants’ first physiological response to water scarcity is stomatal closure, which reduces photosynthetic activity by slowing the rate of carbon dioxide entry into mesophyll cells (Gomez-Candon et al., 2022). The maximum quantum yield of primary photochemistry (*F*v/*F*m) in leaves in the dark-adapted state can reflect wheat’s light-use efficiency under drought conditions (Yu et al., 2021). Reactive oxygen species (ROS) are regarded as one of the mechanisms for enhancing the adaptation of plants to environmental stress conditions (Gill and Tuteja, 2010). The production of ROS can cause chemical damage to DNA and proteins, interfering with a series of physiological and biochemical processes (Cruz de Carvalho, 2008). An active antioxidant system is an important strategy for plants to cope with drought stress, and antioxidant enzymes mainly include superoxide dismutase (SOD), peroxidase (POD), catalase (CAT), and ascorbate peroxidase (APX) (Miller et al., 2010). Another important relevant response of wheat to drought stress is osmoregulation which has long been recognized as an important component of drought tolerance, and osmoregulatory substances include malondialdehyde (MDA), proline (Pro), and soluble proteins (SPC) (Hura et al., 2007; Kumar et al., 2020). Similarly, water stress would significantly impact the endogenous hormonal balance, affecting drought resistance in wheat (Luo et al., 2021). The plant hormone abscisic acid (ABA) plays a key role in regulating drought stress by stimulating short-term responses to maintain water balance by the closing of stomata and inducing the transcription and activity of antioxidant enzymes (Guan et al., 2000; Jiang and Zhang, 2003; Wang et al., 2021). Improved water use efficiency and regulated photosynthetic activity through ABA play a key role in response of plants to drought (Wang et al., 2020; Gouveia et al., 2021).

In recent years, studies have reported some traits associated with drought tolerance in wheat. However, various phenotypic characters, including yield components, plant height, number of tillers, number of spikelets, grain numbers per spike, and thousand-grain weight (Lopes et al., 2012; Zhang et al., 2016; Kadkol et al., 2020; Mdluli et al., 2020; Ahmad et al., 2022), are still the key traits for screening wheat breeding materials (Monneveux et al., 2012). Nakhforoosh et al. (2016) found that dry matter accumulation can be considered a key indicator for screening for drought-resistant cultivars. Lu et al. (2020) found that the relatively low canopy temperature(CT) value at the grain-filling stage and the high chlorophyll content during the late grain-filling stage can be used to screen the winter wheat cultivars adapted to dryland ecosystems. Similarly, at the physiological and biochemical levels, photosynthesis intensity, chlorophyll content, ABA accumulation, antioxidant enzyme activity (SOD, POD, CAT, and APX), and accumulation of osmotic regulatory substances (Pro) can be used as reference indices for drought resistance evaluation (Zivcak et al., 2009; Wang et al., 2014; Song et al., 2017; Ahmed et al., 2019). It is difficult to consider all drought resistance traits when evaluating large populations due to the diversity of drought resistance indicators, and PCA can be used to assess the weight assigned to each indicator and find the principal components that influence all variables (Tahjib-Ul-Arif et al., 2018; Ahmad et al., 2020). In addition, the combination of membership function, cluster analysis, correlation analysis, grey relational analysis, and other methodologies can be used to more accurately and reliably evaluate the performance of plants under adverse conditions (Sun et al., 2021).

There are three main problems with the previous research study. To begin with, some studies have been carried out in greenhouses or pots, where the environment and wheat growth differ from those grown in the field. A single plant or several plants in a greenhouse or pot could not represent the wheat population in the field because wheat is a densely planted crop(Ahmed et al., 2019; Ahmad et al., 2022; Correia et al., 2022). Second, as a result of climate change, drought stress in winter wheat occurs primarily in April and May in the North China Plain, and different study periods result in an inconsistent selection of drought tolerance traits. Finally, most studies considered only the wheat phenotypic traits, while ignoring the effects of photosynthetic, physiological, and biochemical on crop drought resistance (Liu et al., 2016).

Consequently, we cultivated 16 wheat cultivars in a field experiment and measured 24 related traits. The objective of this study was (1) to evaluate the drought resistance of wheat by principal component analysis, membership function, and multivariate statistics, (2) to classify cultivars according to the comprehensive evaluation value, and (3) to screen and evaluate the traits associated with drought resistance in different winter wheat varieties.

## 2 Materials and methods

**Figure 1:**
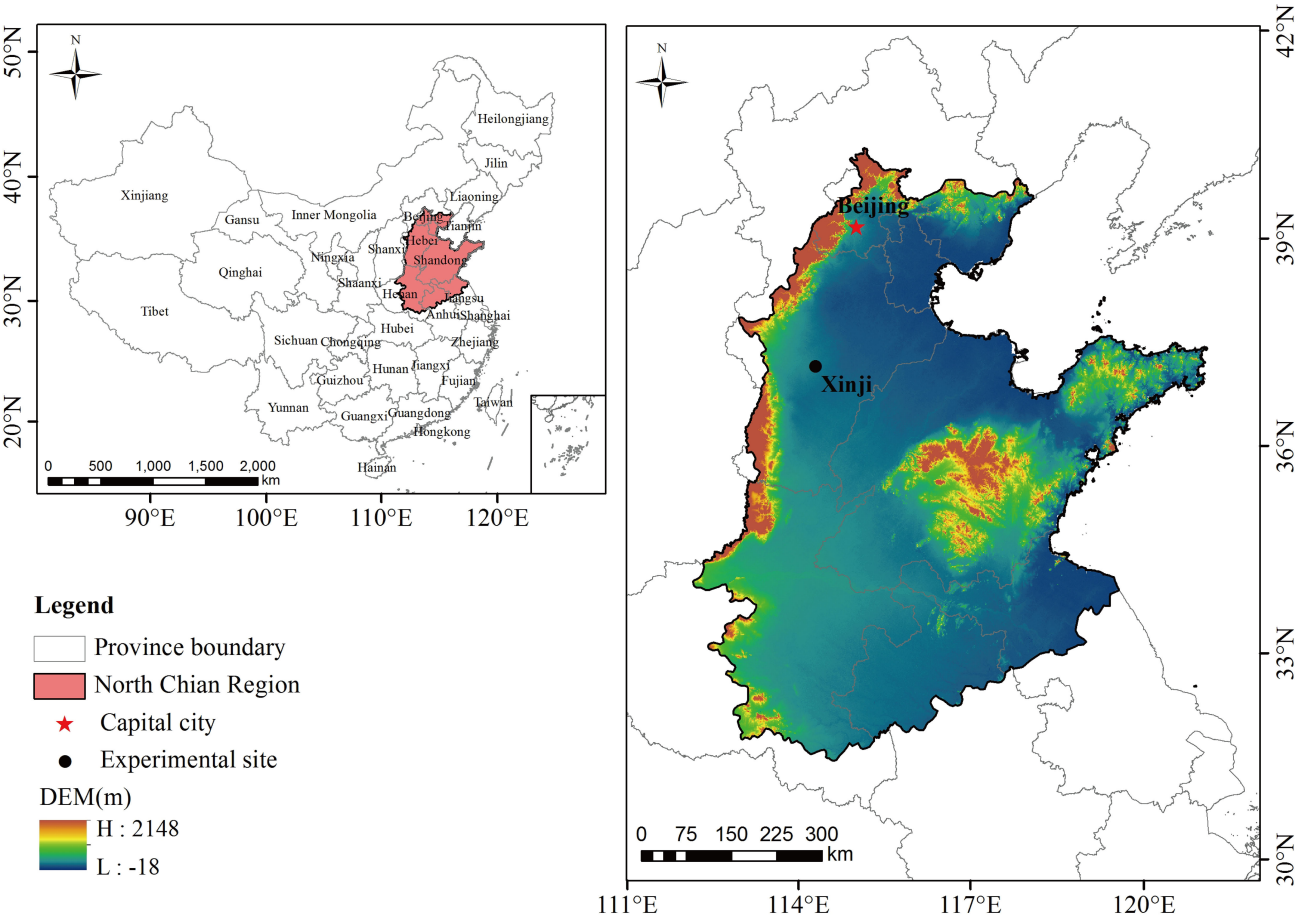
The geographic overview of the North China Plain and the experimental site.

This study was conducted at the Malan Experimental Station (37° 99’N, 115° 20’E), Hebei Agricultural University, from 2019 to 2021 (**Figure 1**). The study area has a sub-humid continental temperate monsoon climate and is located 37 m above sea level. At a depth of 0–200 cm, the average bulk density of soil is 1.56 g/cm^3^, and the field water holding capacity is 22.9%. The 0–40 cm soil layer has a pH of 8.0 and an available phosphorus content of 25.8 mg/kg, a potassium content of 125 mg/kg, and a total nitrogen content of 1.26 g/kg. Before the experiment, the field was planted with summer maize in rotation, and all the maize straw was crushed and returned to the field after harvest.

**Figure 2:**
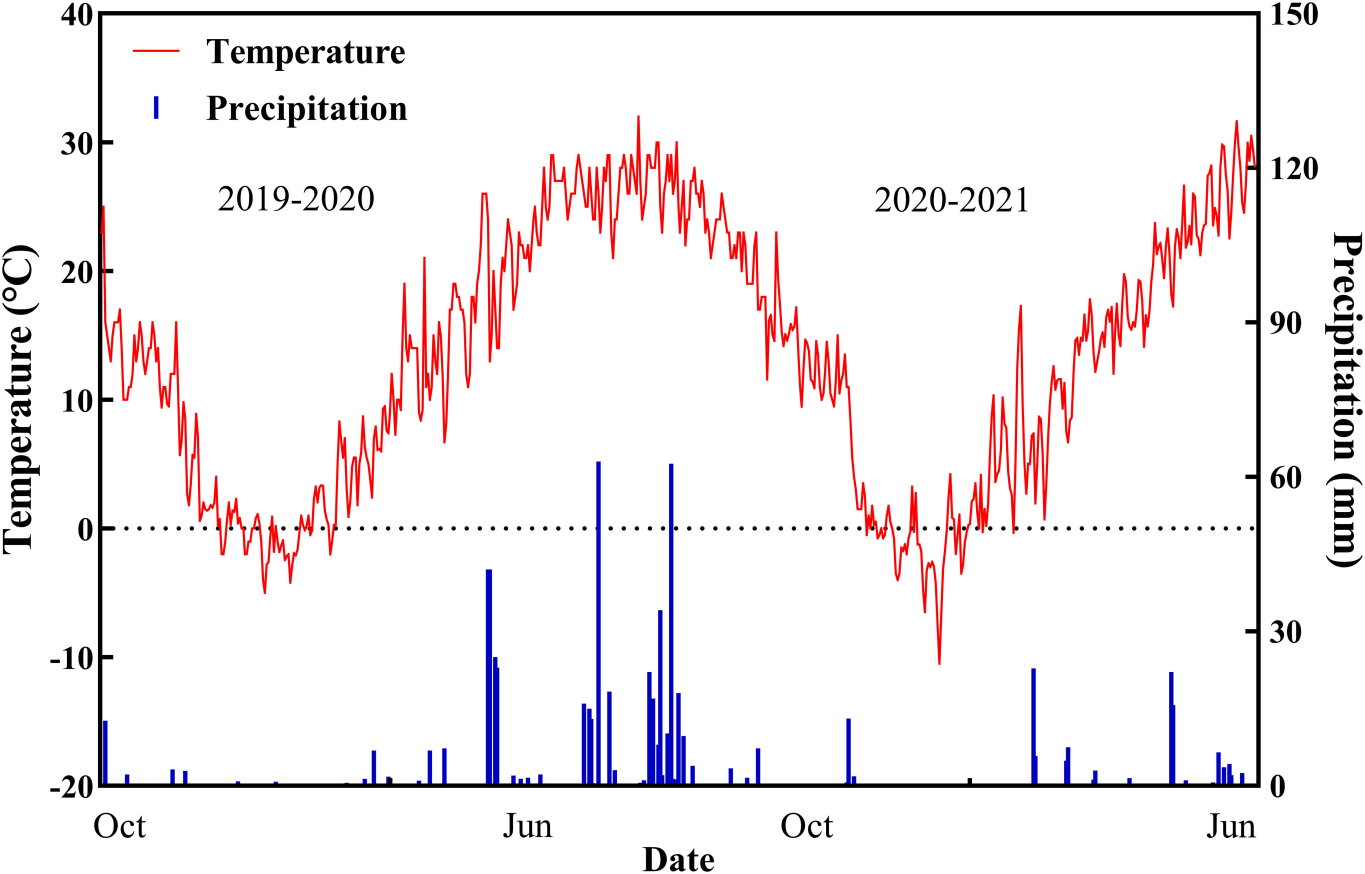
Precipitation and temperature values during the two growing seasons of wheat.

The experiment included two drought treatment variables: normal irrigation (CK, two irrigations of 75 mm each at jointing and anthesis (a total of 150 mm)) and drought stress (DS, no irrigation during the spring) with 16 wheat cultivars (**Table 1**). The Hebei Agricultural University provided all plant materials. We measured 24 drought-tolerant traits in wheat (**Table 2**). The average rainfall during the wheat-growing season has been 124.5 mm, according to historical data recorded by our weather station for nearly 20 years, while the average rainfall for the 2019–2020 and 2020–2021 wheat-growing seasons was 176.2 mm and 103.4 mm, respectively. **Figure 2** depicts the daily temperature and precipitation for 2019–2021. Three replicates for each treatment were arranged in a completely randomized block design. The size of the plot was 66 m^2^ (10 × 6.6 m), and the distance between rows was 15 cm. A total of 240 kg ha^–1^ of nitrogen, 105 kg ha^–1^ of phosphorus, and 120 kg ha^–1^ of potassium fertilizers were applied before planting. No spring topdressing was done during the wheat-growing season. All plots were handled in compliance with prevailing local management standards.

**Table 1:** Names and sources of different wheat cultivars used in this study.

ID	Cultivars	Certification year	Pedigree	ID	Cultivars	Certification year	Pedigree
1	Gaoyou2018(GY2018)	2005	9411/98172	9	Xingmai7(XM7)	2007	935031/GY503
2	Gaoyou5218(GY5218)	2015	XN979/8901–11–14	10	Xingmai13(XM13)	2016	H9117–2/H4589
3	Jimai738(JM738)	2016	G9618/LX99	11	Cangmai6002(CM6002)	1996	LF6154/JM32
4	Shixin828(SX828)	2002	422/SX163/612	12	Hanmai19(HM19)	2018	H02–6018/JM22
5	Shi4366(S4366)	2019	LX99/SY17	13	Shinong086(SN086)	2019	LM14/H6172
6	Jimai585(JM585)	2008	TG genic male sterile population	14	Shimai22(SM22)	2006	L8014/JM38/S4185
7	Jimai418(JM418)	2016	J5157/S20–7221	15	Heng4399(H4399)	2008	H6174/HS28
8	Shiluan02–1(SL02–1)	2007	9411/9430	16	Hengguan35(HG35)	2006	84G749/H87–4263

**Table 2:** Twenty-four drought tolerance traits and their abbreviations.

Trait	Abbreviation	Trait	Abbreviation
Plant height	PH	Transpiration rate	E
Dry matter accumulation	DM	Stomatal conductance	Gs
Spike number	SN	Intercellular CO_2_ concentration	Ci
Grain number	GN	Photochemical efficiency	*F*v/*F*m
Spikelet number per spike	SP	Soluble protein content	SPC
The number of infertile spikelets per spike	SSP	Superoxide dismutase	SOD
Thousand-grain weight	TGW	Peroxidase	POD
Leaf area index	LAI	Catalase	CAT
Canopy temperature	CT	Ascorbate peroxidase	APX
Leaf water content	LWC	Malondialdehyde	MDA
Relative chlorophyll content	SPAD	Proline	Pro
Photosynthetic rate	A	Abscisic acid	ABA

### 2.1 Morphological and yield-associated traits

At 10 days after anthesis, 20 wheat plants were randomly selected from each plot. The plant height (PH, cm), leaf length (*L*, cm), and maximum leaf width (*W*, cm) were measured using a ruler. Leaf area was calculated as follows: $\text{Leaf area}\left(cm^{2}\right)=L\times W\times 0.83$ . Then the leaf area index (LAI) was calculated as follows LAI=Leaf area×N/S : where *N* is the number of wheat plants per field area and *S* is the unit area of the field. Canopy temperature (CT) was measured at noon with a hand-held thermal infrared instrument (FLIR Integrated Imaging Solutions Inc., Canada), with a field of view (FOV) of 25°×20° and a resolution of 320 × 240. Seven days before harvest, plants in 1.2 m^2^ from each plot with three replicates were randomly selected and investigated to determine the spike number (SN). After sampling, the total number of spikelets per spike (SP), the number of infertile spikelets per spike (SSP), and the number of grains per spike (GN) were investigated. Plants with an area of 0.15 m^2^ at ground level were sampled at physiological maturity and oven-dried to a constant weight to determine dry matter accumulation (DMM). Meanwhile, the wheat plants were harvested manually from the plot with a field area of 2 m^2^ (avoiding boundary rows) with three replicates and threshed. The water content was converted to a standard water content of 13% to determine the thousand-grain weight (TGW) and grain yield.

### 2.2 Physiological parameters and hormone contents

The chlorophyll content (SPAD) was measured in 10 flag leaves per plot 10 days after anthesis using a SPAD-502 Minolta chlorophyll meter (Spectrum Technologies, Plainfield, IL, USA). Three points were taken evenly per leaf, and three readings were averaged. As one biological replicate, we take 10 independent flag leaves. At each time point, three biological replicates were collected. All of the samples were immediately frozen in liquid nitrogen and kept at −80°C. The superoxide dismutase(SOD) activity was determined using the nitro-blue tetrazolium (NBT) photoreduction method, while the catalase(CAT) activity was determined spectrophotometrically at 240 nm (Giannopolitis and Ries, 1977). The guaiacol chromogenic method was used to determine the peroxidase (POD) activity (Han et al., 2008). The malondialdehyde (MDA) contents were determined by thiobarbituric acid and sulfosalicylic acid dihydrate methods (Hodges et al., 1999). Soluble protein content (SPC) was determined by the Coomassie brilliant blue method (Campion et al., 2011). The ascorbate peroxidase(APX) activity was determined using the method of Madhusudhan (2003) (Madhusudhan et al., 2003). The proline(Pro) content was quantified using the ninhydrin colorimetric method (Magne and Larher, 1992). Abscisic acid (ABA) was determined by high-performance liquid chromatography (Benech Arnold et al., 1991).

### 2.3 Leaf gas exchange parameters

The LI-6800 Portable Photosynthesis System (Li-cor, Lincoln, NE, USA) was used to measure leaf gas exchange 10 days after anthesis. The leaf chamber was maintained under control, with a reference CO_2_ concentration of 400 µ mol mol^−1^, a leaf temperature of 25 °C, a saturated photosynthetic photon flux density (PPFD) of 1000 µ mol m^–2^ s^−1^, and a relative humidity of 60%-70%. In the morning, at 09:00–11:00, gas exchange was measured three times in the flag leaves with the same size and orientation in plants from each plot. Net photosynthetic rate (A), stomatal conductance (Gs), transpiration rate (Tr), and intercellular CO_2_ concentration (Ci) were measured. A portable FMS-2 chlorophyll fluorometer (Hansatech, King’s Lynn, UK) was used to measure the chlorophyll fluorescence parameter *F*v/*F*m in flag leaves at 0:00–2:00.

### 2.4 Analysis of adaptation to drought


(1) $$DC=\frac{X_{DS}}{X_{CK}}$$

(2) $$U\left(X_{j}\right)=\frac{X_{j}-X_{min}}{X_{max}-X_{min}}$$

(3) $$W_{j}=\frac{P_{j}}{\sum_{j=1}^{n}P_{j}}$$

(4) $$D=\sum_{i=1}^{n}\left[u\left(X_{j}\right)\times Wj\right]\;j=\;1,2,\ldots ,n$$

DC is the drought-tolerant coefficient, whereas $X_{DS}$ and $X_{CK}$ are the trait values for cultivars evaluated under drought stress (DS) and normal irrigation (CK) conditions, respectively. U is the membership function value of drought tolerance based on the trait of genotypes, while $X_{j}$ is the jth composite indicator, and $X_{max}$ and $X_{min}\;$ are the maximum and minimum values of the jth composite index, respectively. $W_{j}$ is the weight of the jth comprehensive index, and $\;P_{j\;}$ is the variance contribution rate of the jth comprehensive index. The membership function value (U) of the comprehensive index of each wheat variety was calculated by the formula (2). The contribution rate of each comprehensive index was used to calculate the weight of principal components, and the index weight and membership function value were used to calculate the comprehensive evaluation value of drought resistance (D value). The D value is the comprehensive evaluation value of each genotype’s drought resistance, as determined by a comprehensive evaluation of the index of various wheat cultivars under drought stress.


(5) $$LO_{i}\left(k\right)=(\Delta_{min}+\rho \Delta_{max})/\left(\Delta O_{i}\left(k\right)\right)+\rho \Delta_{max})$$

(6) $$\gamma O_{i}\left(k\right)=\;1/n\sum_{k=1}^{n}LO_{i}\left(k\right)$$

Where $LO_{i}\;\left(k\right)$ is the grey correlation coefficient and $\gamma O_{i}\left(k\right)$ is the grey relational degree. In the formula, $\Delta O_{i}\;\left(k\right)$ is the absolute difference between the two sequences at time k . $\Delta_{min}$ and $\Delta_{max}$ are the minimum and maximum values of the absolute difference between all compared sequences at each time; $\Delta_{min}=\;0$ ; and the resolution coefficient ρ= 0.5 .

### 2.5 Data analysis

Twenty-four traits were used for analysis. Data were summarized and analyzed using Excel 2016 (Microsoft, Redmond, WA, USA), with measurements for each trait corresponding to three independent replicates. SPSS 21.0 (SPSS Inc., Chicago, IL, USA) was used to analyze variance, principal component analysis, and stepwise regression analysis. The images were drawn using ArcGIS 10.2, Origin 2021 (OriginLab Corporation, Northampton, MA, USA), and GraphPad Prism 9.0 (GraphPad Software, Inc., San Diego, CA, USA).

## 3 Results

### 3.1 Response of wheat yield to drought stress

**Figure 3:**
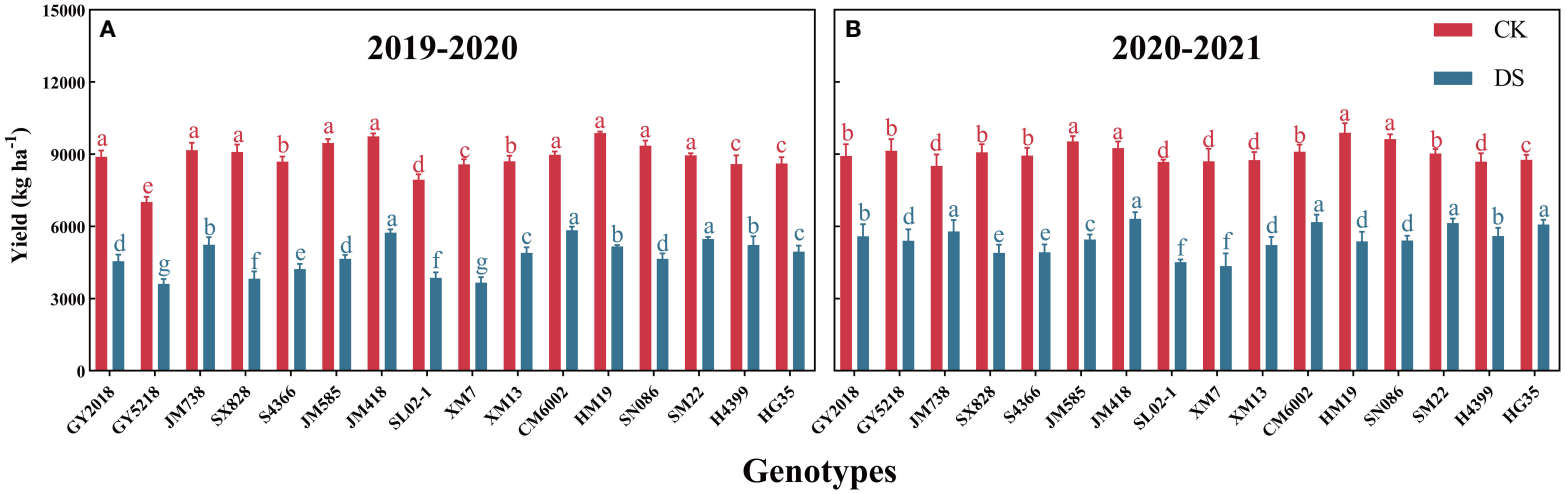
Yield of 16 wheat cultivars grown under CK and DS conditions during 2019-2020 **(A)** and 2020-2021 **(B)**; Data are represented as mean ±SD (n = 3). Different lowercase letters on columns indicate significant differences between the means of the same irrigation treatments 5% level by LSD test.

**Figure 3** depicts the yield performance of 16 wheat cultivars in two growing seasons under normal irrigation (CK) and drought stress (DS) conditions. There were significant yield differences between irrigation treatments and cultivars, which were more noticeable in 2019–2020 (**Figure 3A**) than in 2020–2021. (**Figure 3B**). During the two wheat-growing seasons, HM19, JM585, JM418, and SN086 showed a higher average yield (9492.6–9887.5 kg ha^–1^) under CK, while GY5218 and SL02–1 showed a lower average yield (8083.8–8309.1 kg ha^–1^). Under DS conditions, JM418, CM6002, and H4399 all had higher average yields (5807.95–6032.7 kg ha^–1^), while SX828, SL02–1, and XM7 had lower average yields (4009.75–4367.3 kg ha^–1^). Compared with CK, CM6002, SM22, JM418, and HG35 had the lowest average decrease (by 33.5%) under DS within two years, while XM7 and SX828 showed the biggest declines in production (53.6% and 52%, respectively).

### 3.2 Values of each trait and drought tolerant coefficient under two water conditions

**Figure 4:**
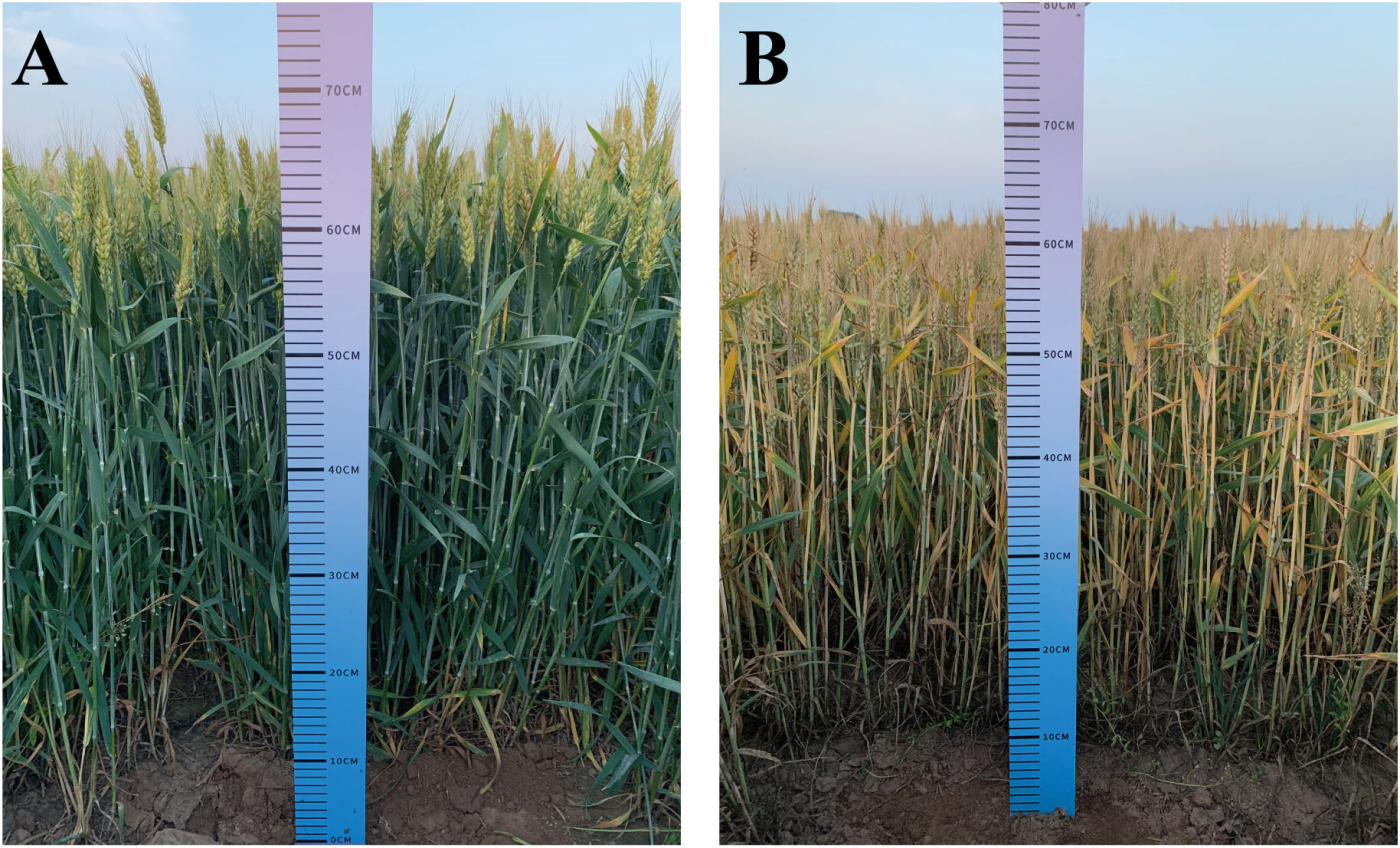
The growth of winter wheat in the field under normal irrigation **(A)** and drought stress **(B)**.

To determine the effect of drought on different wheat cultivars, we investigated 24 morphological traits, photosynthetic traits, physiological indices, and yield component parameters related to drought tolerance in wheat after anthesis and at maturity for two consecutive years (**Table 3**). **Figure 4** depicts the growth of wheat in the field under various treatments. The findings revealed that all traits varied with irrigation supply. (P≤0.05). SSP, CT, SPC, MDA, Pro, and ABA in the DS treatment had significantly higher values than those in the CK treatments (Drought resistance coefficient>1). However, the mean values of the other 18 traits for the DS treatment were lower than those under CK (Drought resistance coefficient<1). The degree of decline in treatments from CK to DS also differed in the two growing seasons. The average coefficient of variation (CV) for the measured traits was 6.4 under CK but 9.0 under DS. The average drought tolerant coefficient of the two wheat-growing seasons ranged from 0.43 to 2.02, and the CV ranged from 0.66% to 19.62%. The range of variation for every single trait was different. Therefore, the drought tolerance of wheat was evaluated by the drought-tolerant coefficient of different characters, and the results were different.

**Table 3:** Descriptive statistics of the various traits investigated under normal (CK) and drought stress (DS) conditions in 2 growing seasons of wheat.

Year	2019–2020						2020–2021					
Treatment	CK		DS		Drought resistance coefficient		CK		DS		Drought resistance coefficient	
Statistical parameter	Mean	CV (%)	Mean	CV (%)	Mean	CV (%)	Mean	CV (%)	Mean	CV (%)	Mean	CV (%)
PH	76.75^a^	6.32	64.80^b^	5.54	0.84	2.90	79.41^a^	6.65	65.42^b^	6.81	0.83	3.14
DM	18811.75^a^	5.72	10354.89^b^	16.44	0.55	13.69	19557.09^a^	4.99	11521.66^b^	9.00	0.59	10.63
SN	56.73^a^	10.61	41.71^b^	11.87	0.74	7.45	47.49^a^	9.10	34.36^b^	9.04	0.72	3.60
GN	34.44^a^	4.68	26.94^b^	7.73	0.78	6.64	35.78^a^	3.14	28.01^b^	4.49	0.78	4.61
SP	16.51^a^	4.32	13.60^b^	7.10	0.82	6.83	16.48^a^	5.15	13.39^b^	5.53	0.81	2.42
SSP	1.93^b^	18.40	3.91^a^	14.07	2.02	19.62	2.44^b^	20.09	4.49^a^	10.35	1.90	18.82
TGW	40.38^a^	7.29	36.60^b^	7.97	0.91	3.55	36.30^a^	6.07	33.76^b^	6.42	0.93	2.81
LAI	7.40^a^	4.52	4.56^b^	10.29	0.62	9.17	6.30^a^	4.67	4.12^b^	9.30	0.65	7.74
CT	23.12^b^	1.49	25.07^a^	3.32	1.08	2.41	25.16^a^	1.77	26.95^a^	3.40	1.07	2.13
LWC	73.03^a^	2.66	67.43^b^	2.97	0.92	2.47	67.11^a^	2.03	61.67^b^	1.83	0.92	1.61
SPAD	57.69^a^	2.35	47.18^b^	7.79	0.82	7.74	57.43^a^	2.65	48.39^b^	5.56	0.84	4.98
A	26.20^a^	5.92	21.16^b^	7.83	0.81	6.42	26.10^a^	4.63	20.44^b^	6.16	0.78	5.11
E	4.49^a^	9.80	3.26^b^	11.24	0.73	8.97	4.33^a^	6.06	3.28^b^	7.40	0.76	6.61
Gs	377.91^a^	3.66	323.89^b^	3.53	0.86	4.32	376.23^a^	2.51	323.93^b^	4.79	0.86	3.54
Ci	333.43^a^	1.97	276.02^b^	7.09	0.83	6.55	333.30^a^	1.40	288.30^b^	3.90	0.86	3.66
*F*v/*F*m	0.84^a^	0.55	0.81^b^	1.18	0.97	0.99	0.84^a^	0.53	0.81^b^	0.82	0.97	0.66
SPC	48.81^b^	7.26	71.37^a^	10.42	1.46	10.10	57.88^b^	4.59	84.37^a^	7.05	1.46	8.29
SOD	531.65^a^	4.35	385.97^b^	11.32	0.73	9.23	572.08^a^	6.36	412.47^b^	8.07	0.72	8.27
POD	251.18^a^	7.47	161.92^b^	15.52	0.65	14.52	250.21^a^	9.76	153.70^b^	15.68	0.62	17.28
CAT	131.42^a^	2.52	78.94^b^	11.83	0.60	13.38	141.78^a^	14.95	61.63^b^	25.40	0.43	16.18
APX	6.30^a^	7.10	3.80^b^	16.17	0.60	14.36	6.53^a^	6.14	5.02^b^	8.37	0.77	6.99
MDA	30.76^b^	11.79	51.92^a^	14.53	1.69	12.59	34.59^b^	15.65	55.84^a^	18.23	1.61	8.18
Pro	31.48^b^	10.84	46.18^a^	11.62	1.47	15.59	33.57^b^	11.18	51.42^a^	15.43	1.53	8.79
ABA	32.50^b^	7.19	53.70^a^	13.22	1.65	12.27	35.16^b^	8.08	55.08^a^	6.03	1.58	10.36

Plant height (PH), dry matter accumulation (DM), spike number (SN), grain number (GN), spikelet number per spike (SP), the number of infertile spikelets per spike (SSP), thousand-grain weight (TGW), leaf area index (LAI), canopy temperature (CT), leaf water content (LWC), relative chlorophyll content (SPAD), photosynthetic rate (A), transpiration rate (E), stomatal conductance (Gs), intercellular CO_2_ concentration (Ci), photochemical efficiency (Fv/Fm), soluble protein content (SPC), superoxide dismutase (SOD), peroxidase (POD), catalase (CAT), ascorbate peroxidase (APX), malondialdehyde (MDA), proline (Pro), abscisic acid (ABA), normal irrigation (CK), drought stress (DS), and the coefficient of variation (CV); different letters denote significant differences between means of CK and DS treatments at a significance level of 0.05 (P< 0.05) by paired sample t-test.

### 3.3 Cluster analysis and correlation analysis of each trait

**Figure 5:**
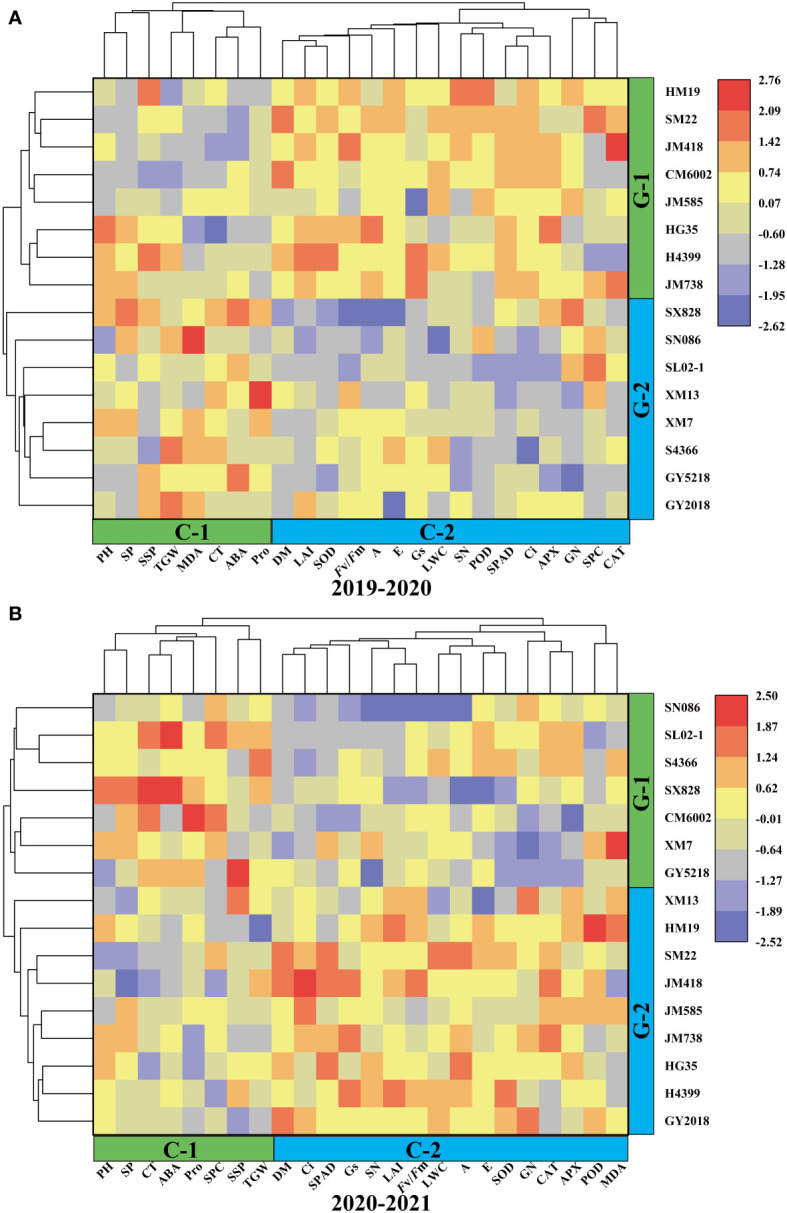
Hierarchical clustering and heatmaps based on correlationsbetween drought tolerant coefficients for 16 wheat cultivars and 24 different traits during 2019–2020 **(A)** and 2020–2021 **(B)**.

Wheat traits were divided into two clusters using the clustering analysis of cultivars based on the drought-tolerant coefficient for two years (**Figure 5**). Cluster-1(C1) and Cluster-2(C2) comprised 8 and 16 traits, respectively, during the two wheat-growing seasons, and the highly correlated traits were assembled into a population. During 2019–2020, PH, SP, SSP, TGW, MDA, CT, ABA, and Pro were separated from the other parameters (**Figure 5A**). During 2020–2021, however, SP, CT, ABA, Pro, SPC, SSP, and TGW were separated from the other traits (**Figure 5B**). Based on the drought-tolerant coefficient of each trait, 16 wheat cultivars were included in group-1(G1) and group-2(G2), and cultivars in the same group showed similar drought tolerance. In two years, HM19, SM22, JM418, JM515, HG35, H4399, and JM738 were divided into a same group, while SX828, SN086, SL02–1, XM7, S4366, and GY5218 formed another group. The drought-tolerant coefficients of different traits in the same cluster and drought tolerance among cultivars showed similarity, proving that this grouping method was representative.

**Figure 6:**
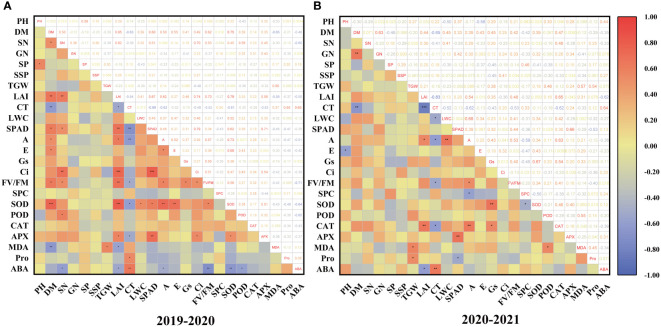
Distribution and correlation among drought tolerant coefficient of each character of wheat cultivars during 2019–2020 **(A)** and 2020–2021 **(B)**; *, **, and *** show significant differences between means of different treatment groups at 0.05, 0.01, and 0.001 significance levels, respectively.

Spearman’s correlation analysis revealed that the correlation between traits was similar in two years (**Figure 6**). LAI was correlated positively with SN and *F*v/*F*m. CT was positively correlated with ABA but negatively with DM, SPAD, and A. SOD was correlated positively with A and E. The correlation between these traits also indicates the overlap of information shared by them. These findings demonstrated that drought tolerance in wheat is a complex trait, and evaluating drought resistance in wheat based on a single trait is insufficient. Therefore, it is necessary to use the multivariate statistical method for further analysis with multiple indicators.

### 3.4 Comprehensive evaluation and screening of drought tolerance traits using PCA of drought resistance traits of wheat cultivars

Further PCA of the drought tolerant coefficient of 24 tested traits showed that seven principal components were extracted in the two growing seasons (**Tables 4**, **5**). From 2019 to 2020, the variance contribution rates of CI comprehensive indices (from CI_1_ to CI_7_) were 34.20%, 11.80%, 10.24%, 8.25%, 7.29%, 5.74%, and 4.94%, respectively. The cumulative contribution rate was 82.44%. From 2020 to 2021, however, the variance contribution rates of CI_1_ to CI_7_ were 31.79%, 12.88%, 11.58%, 10.38%, 8.15%, 5.53%, and 4.36%, respectively, with the cumulative contribution rate of 84.65%. The original 24 single traits could be converted into 7 new independent comprehensive indices, which can cover most of the information. In both wheat-growing seasons, the first factors contributed more than 30%. From 2019 to 2020, the load coefficients of POD, CT, A, SOD, and SPAD were large. However, from 2020 to 2021, the load coefficients of SPAD, CT, A, Pro, and Ci were large. These traits mainly reflect the information regarding stress tolerance, canopy parameters, and photosynthetic physiological parameters of winter wheat. PCA could fully reflect the primary and secondary functions of indicators in wheat screened for drought tolerance to comprehensively evaluate the drought tolerance differences between different cultivars of wheat.

**Table 4:** Component matrix and the cumulative contribution rate of principal components during 2019–2020 and 2020–2021.

Principle factor	2019–2020							2020–2021						
	CI_1_	CI_2_	CI_3_	CI_4_	CI_5_	CI_6_	CI_7_	CI_1_	CI_2_	CI_3_	CI_4_	CI_5_	CI_6_	CI_7_
Factor weight	0.415	0.143	0.124	0.100	0.088	0.070	0.060	0.376	0.152	0.137	0.123	0.096	0.065	0.051
Eigenvalue	8.208	2.831	2.458	1.979	1.749	1.377	1.185	7.630	3.091	2.778	2.490	1.956	1.327	1.045
CR (%)	34.198	11.795	10.242	8.245	7.286	5.738	4.937	31.790	12.879	11.577	10.375	8.148	5.529	4.356
CCR (%)	34.198	45.993	56.235	64.480	71.766	77.504	82.441	31.790	44.669	56.246	66.621	74.769	80.298	84.654
Eigenvector														
PH	0.168	0.231	-0.260	0.137	0.805	-0.036	-0.107	0.070	-0.006	0.107	-0.073	0.181	0.878	0.104
DM	0.331	0.496	0.454	0.370	-0.002	0.037	0.347	0.433	0.127	0.172	0.513	0.210	-0.348	0.451
SN	0.193	0.484	0.683	0.044	-0.046	-0.002	-0.370	0.299	0.463	0.567	-0.201	0.099	0.371	0.136
GN	0.223	0.231	0.111	-0.821	-0.030	-0.132	-0.250	-0.060	0.085	-0.104	0.108	0.859	-0.058	0.225
SP	-0.125	-0.182	-0.001	-0.128	0.849	0.118	-0.014	-0.243	-0.019	-0.177	-0.101	-0.106	0.833	-0.246
SSP	-0.041	0.018	-0.130	0.024	-0.050	-0.180	0.771	0.291	0.514	-0.188	0.344	0.032	0.506	-0.279
TGW	0.134	-0.813	-0.153	0.275	0.211	0.082	-0.065	0.128	-0.846	-0.124	-0.214	-0.003	-0.174	-0.182
LAI	0.581	0.533	0.258	0.384	0.052	0.088	-0.222	0.151	0.389	0.763	0.076	0.135	-0.086	0.414
CT	0.754	0.144	0.307	0.177	0.163	-0.039	0.072	0.712	0.344	0.220	0.169	0.282	-0.226	0.214
LWC	0.187	0.469	0.053	0.109	-0.492	0.486	0.238	0.277	0.034	0.544	0.540	-0.363	0.198	0.071
SPAD	0.635	0.436	0.240	0.030	0.074	0.388	-0.116	0.897	0.024	0.192	0.095	0.070	0.057	0.250
A	0.733	0.069	-0.047	0.519	-0.143	0.042	0.275	0.562	0.089	0.676	0.303	0.116	0.030	-0.053
E	0.330	0.140	0.345	0.180	-0.199	0.097	0.630	0.032	0.124	0.102	0.871	-0.038	-0.100	-0.123
Gs	0.196	0.150	-0.073	0.696	-0.031	0.294	-0.074	0.399	-0.024	0.223	0.101	0.022	0.311	0.717
Ci	0.460	0.588	0.306	0.080	-0.224	0.152	-0.355	0.457	0.281	0.147	0.068	0.188	-0.241	0.462
*F*v/*F*m	0.308	0.310	0.417	0.698	-0.057	-0.212	-0.021	0.034	0.024	0.859	0.192	-0.001	-0.200	0.279
SPC	0.264	0.013	-0.053	0.165	-0.022	0.813	-0.179	0.098	0.096	0.254	-0.216	0.141	-0.212	0.804
SOD	0.656	0.290	0.373	0.284	0.075	0.220	0.305	0.149	0.080	0.453	0.716	0.423	0.019	-0.008
POD	0.157	0.083	0.879	-0.273	-0.256	0.083	0.044	0.226	0.879	0.084	-0.105	-0.075	-0.168	0.131
CAT	0.501	0.193	0.015	-0.130	-0.312	-0.617	0.025	0.388	-0.136	0.083	0.398	0.557	-0.014	-0.203
APX	0.536	0.479	0.202	-0.281	0.314	0.308	-0.068	0.139	-0.144	0.278	-0.121	0.839	0.210	-0.033
MDA	0.105	0.837	-0.015	0.292	0.157	-0.040	0.158	0.162	-0.559	-0.093	0.577	0.071	-0.254	0.300
Pro	0.835	-0.175	0.115	-0.184	-0.194	0.083	-0.053	0.522	0.022	-0.078	0.014	0.672	0.025	0.224
ABA	0.528	0.007	0.698	0.226	-0.003	-0.249	0.047	0.397	0.656	0.280	0.188	-0.046	-0.426	-0.133

CI, comprehensive indices; CR, Contribution ratio; CCR, Cumulative contribution ratio.

**Table 5:** The evaluation of drought tolerance, comprehensive indices, and D value of different wheat cultivars during 2019–2020.

Cultivar	CI_1_	CI_2_	CI_3_	CI_4_	CI_5_	CI_6_	CI_7_	*u*(*X*_1_)	*u*(*X*_2_)	*u*(*X*_3_)	*u*(*X*_4_)	*u*(*X*_5_)	*u*(*X*_6_)	*u*(*X*_7_)	D-value	Rank
GY2018	2.544	3.582	38.616	-2.048	22.146	-14.122	-1.672	0.563	0.587	0.866	0.341	0.572	0.396	0.378	0.560	5
GY5218	-0.795	-15.873	36.754	-3.766	67.144	43.942	1.205	0.372	0.242	0.853	0.237	0.825	1.000	0.577	0.496	9
JM738	-0.516	-5.420	-45.112	2.396	-4.503	12.509	-6.060	0.388	0.427	0.286	0.611	0.423	0.673	0.074	0.407	10
SX828	-3.570	-29.575	-86.504	-5.518	98.285	-7.207	-6.947	0.213	0.000	0.000	0.130	1.000	0.468	0.012	0.223	16
S4366	-6.228	8.421	12.143	-0.558	13.791	43.966	0.575	0.061	0.672	0.683	0.431	0.525	1.000	0.533	0.398	11
JM585	-1.980	2.150	26.398	-0.613	18.472	23.185	6.701	0.304	0.561	0.781	0.428	0.552	0.784	0.958	0.507	8
JM418	5.159	26.932	57.984	6.617	-55.849	-37.040	6.792	0.712	1.000	1.000	0.867	0.134	0.157	0.964	0.730	1
SL02–1	-3.858	-26.321	51.913	-7.664	55.969	28.498	6.702	0.197	0.058	0.958	0.000	0.762	0.839	0.958	0.392	12
XM7	-4.772	6.647	-48.544	0.176	23.305	-1.518	-4.277	0.144	0.641	0.263	0.476	0.579	0.527	0.197	0.332	13
XM13	-6.470	-9.993	-29.628	-1.766	-6.129	4.875	-2.525	0.047	0.347	0.394	0.358	0.414	0.593	0.319	0.251	14
CM6002	-1.174	8.154	35.107	8.806	-48.981	-19.036	2.276	0.350	0.668	0.842	1.000	0.173	0.344	0.651	0.524	7
HM19	9.374	7.010	40.231	-0.627	-30.028	-52.144	3.087	0.953	0.647	0.877	0.427	0.279	0.000	0.707	0.707	2
SN086	-7.298	-6.359	-30.361	-7.184	14.492	16.589	0.816	0.000	0.411	0.389	0.029	0.529	0.715	0.550	0.239	15
SM22	5.557	8.422	41.293	5.502	-79.779	-34.760	7.312	0.735	0.672	0.884	0.799	0.000	0.181	1.000	0.663	3
H4399	10.187	5.486	-33.865	3.149	-43.342	-14.601	-7.126	1.000	0.620	0.364	0.657	0.205	0.391	0.000	0.660	4
HG35	3.840	16.738	-66.427	3.099	-44.997	6.864	-6.859	0.637	0.820	0.139	0.654	0.195	0.614	0.018	0.525	6
W*_j_*								0.415	0.143	0.124	0.100	0.088	0.070	0.060		

**Figure 7:**
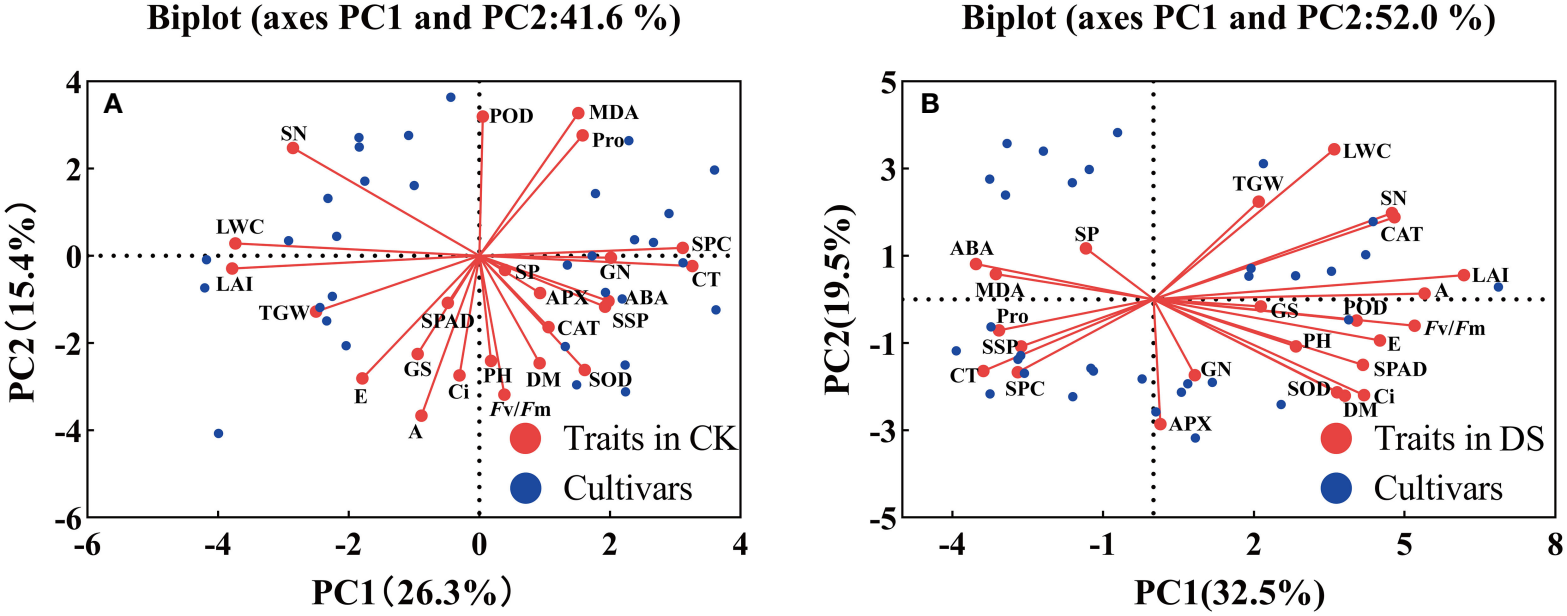
Biplot of 24 different traits of 16 wheat cultivars under CK **(A)** and DS **(B)** treatments.

In this experiment, 24 traits were evaluated by PCA under two moisture conditions, and the biplot showed the distribution of 16 wheat cultivars along the axes for factor 1 and factor 2 in each corner of the scatter plot. The contribution rate of the first two principal components was 41.6% under the CK treatment (**Figure 7A**), with PC 1 explaining 26.3% and PC 2 explaining 15.4% of the total variation. Vectors that were parallel or close to each other showed a strong positive correlation between traits. In contrast, vectors that were in opposite directions (at 180°) showed a high negative correlation, and vectors that were laterally oriented showed a weak correlation. PC1 was mostly represented by CT, SPC, LAI, and LWC, while PC2 was mostly represented by MDA, POD, A, and *F*v/*F*m. Under the DS treatment (**Figure 7B**), however, the contribution rate of the first two principal components was 52.0%, of which PC 1 explained 32.5% but PC 2 explained 19.5%. PC1 was characterized by LAI, A, and *F*v/*F*m, while PC2 was mainly characterized by LWC and APX. To evaluate the difference in drought resistance among 16 wheat cultivars, PCA could fully reflect the primary and secondary effects of indices screened for wheat drought resistance. In general, the PCA biplot could explain the relationship between each index and the contribution of each trait to the principal component under various moisture conditions.

### 3.5 The comprehensive evaluation of drought-tolerant cultivars

According to formula (2), the membership function values of each comprehensive index for various cultivars were determined (**Tables 6**, **7**). In the principal component, the higher the CI value, the stronger the drought resistance of the cultivar. In CI_1_, for example, H4399 had the highest u (*X*_1_) value (1.000) during 2019–2020, indicating that the cultivar had the highest significant drought resistance among CI_1_, whereas SN086 had the lowest µvalue (*X*_1_) (0.000), indicating the cultivar had the lowest drought resistance. JM418 had the highest u (*X*_1_) value in 2020–2021, while SX828 had the lowest. The weight of each comprehensive index was calculated using formula (3). From 2019 to 2020, the weights of the seven comprehensive indices (from CI_1_ to CI_7_) were 0.415, 0.143, 0.124, 0.100, 0.088, 0.070, and 0.060, respectively. From 2020 to 2021, however, the weights of these indices were 0.376, 0.152, 0.137, 0.123, 0.096, 0.065, and 0.051, respectively.

**Table 6:** The evaluation of drought tolerance, comprehensive indices, and D value of different wheat cultivars during 2020–2021.

Cultivar	CI_1_	CI_2_	CI_3_	CI_4_	CI_5_	CI_6_	CI_7_	*u*(*X*_1_)	*u*(*X*_2_)	*u*(*X*_3_)	*u*(*X*_4_)	*u*(*X*_5_)	*u*(*X*_6_)	*u*(*X*_7_)	D-value	Rank
GY2018	0.817	4.499	-0.019	6.961	19.470	6.586	-1.314	0.504	0.617	0.425	0.871	0.597	0.906	0.327	0.582	6
GY5218	-3.765	11.670	-2.015	-4.688	-19.990	-3.214	11.204	0.356	0.706	0.252	0.365	0.312	0.128	0.964	0.408	11
JM738	-0.818	-11.530	0.205	9.927	47.475	1.435	-2.461	0.451	0.419	0.445	1.000	0.800	0.497	0.268	0.540	7
SX828	-14.732	-45.354	-4.833	-11.453	38.889	-4.497	9.496	0.000	0.000	0.007	0.071	0.738	0.026	0.877	0.128	16
S4366	5.646	-10.808	-0.341	-5.031	-39.914	0.003	-7.734	0.661	0.428	0.397	0.350	0.169	0.384	0.000	0.452	9
JM585	-5.080	1.828	2.101	-0.006	60.214	-1.769	-1.424	0.313	0.584	0.609	0.568	0.892	0.243	0.321	0.478	8
JM418	16.090	20.579	5.688	8.202	-63.244	-4.831	-2.344	1.000	0.816	0.920	0.925	0.000	0.000	0.274	0.753	1
SL02–1	0.556	-3.682	-3.867	-4.216	-42.756	-1.426	-6.023	0.496	0.516	0.091	0.385	0.148	0.270	0.087	0.361	12
XM7	-5.366	-13.013	0.703	-6.139	20.278	4.280	11.912	0.304	0.400	0.488	0.302	0.603	0.723	1.000	0.435	10
XM13	-10.673	21.697	-2.651	4.165	-35.153	-1.407	3.286	0.132	0.830	0.197	0.750	0.203	0.272	0.561	0.361	13
CM6002	-2.260	-0.934	-1.367	-13.081	-3.184	-3.023	1.733	0.405	0.550	0.308	0.000	0.434	0.143	0.482	0.354	14
HM19	8.822	-10.533	6.605	-2.651	9.795	-2.445	-2.266	0.764	0.431	1.000	0.453	0.528	0.189	0.278	0.622	4
SN086	-13.772	0.362	-4.916	-0.259	75.198	-3.892	1.761	0.031	0.566	0.000	0.557	1.000	0.075	0.483	0.292	15
SM22	5.398	35.438	2.913	7.207	-9.909	1.732	-6.527	0.653	1.000	0.680	0.882	0.385	0.521	0.061	0.673	2
H4399	11.374	3.115	1.331	4.357	-30.351	7.767	-5.265	0.847	0.600	0.542	0.758	0.238	1.000	0.126	0.671	3
HG35	7.767	-3.336	0.463	6.708	-26.817	4.701	-4.034	0.730	0.520	0.467	0.860	0.263	0.757	0.188	0.607	5
W*_j_*								0.376	0.152	0.137	0.123	0.096	0.065	0.051		

**Table 7:** The grey correlation degree between the drought tolerant coefficient and the D value of each trait.

Traits	Correlation degree	Rank	Traits	Correlation degree	Rank
ABA	0.685	1	Pro	0.655	13
A	0.669	2	SPAD	0.653	14
PH	0.666	3	SOD	0.65	15
GN	0.664	4	CAT	0.649	16
TGW	0.663	5	E	0.648	17
MDA	0.663	6	SSP	0.648	18
Gs	0.662	7	APX	0.646	19
LWC	0.661	8	LAI	0.645	20
CT	0.661	9	SN	0.641	21
Ci	0.659	10	SPC	0.634	22
FV/FM	0.658	11	DM	0.62	23
SP	0.657	12	POD	0.605	24

**Figure 8:**
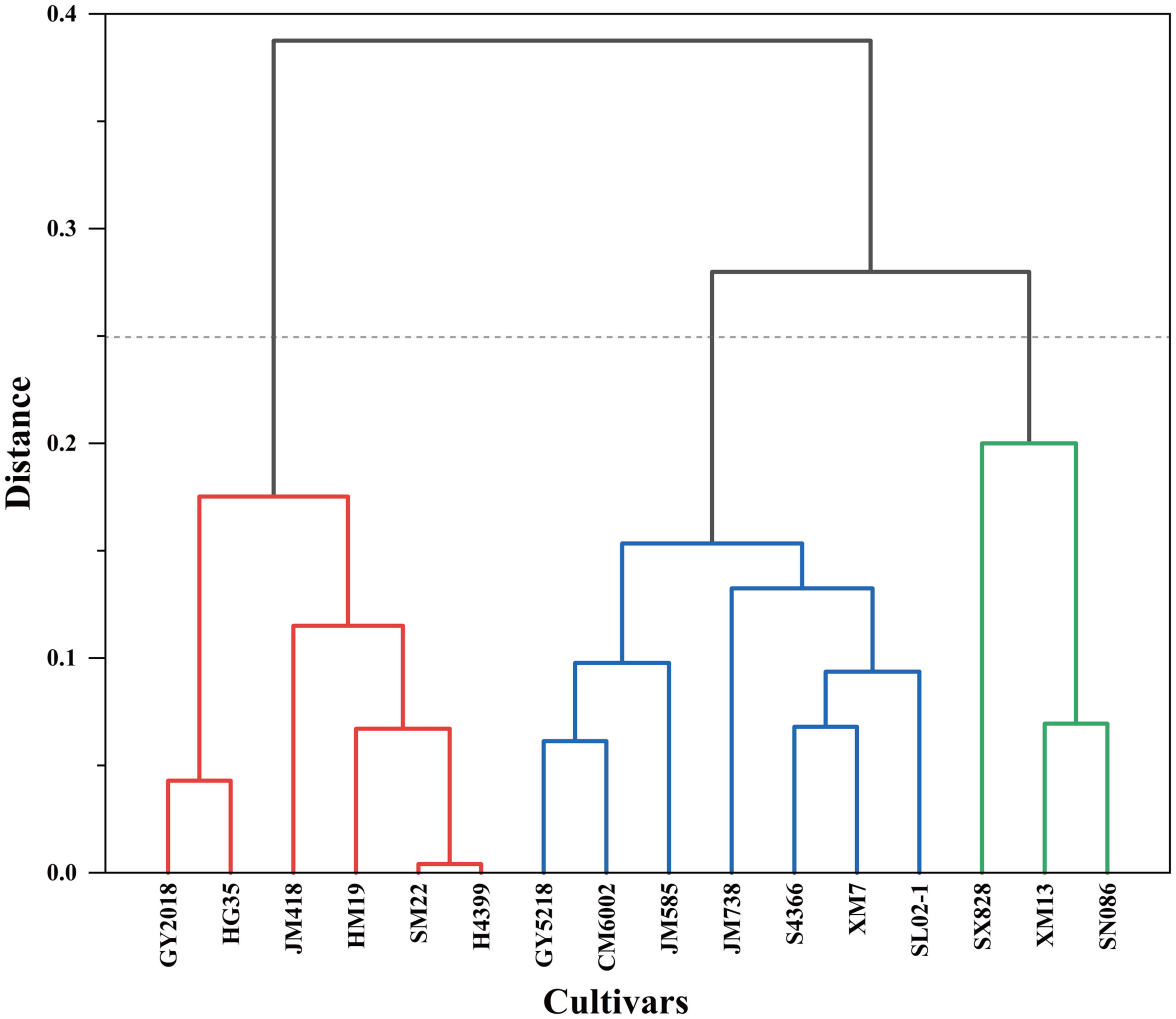
Systematic clustering of 16 wheat cultivars based on D-values.

The comprehensive evaluation value of drought tolerance (D value) was calculated by the formula (4), and the drought tolerance of wheat cultivars was ranked according to the D value. The lower the D value, the more sensitive the wheat crop to drought stress, and the lower the drought tolerance. In 2019–2020, JM418 had the highest D value (0.730), followed by HM19, SM22, and H4399 (**Table 4**). The D value of SX828 was the lowest (0.223), indicating that it was less tolerant to drought stress. In 2020–2021, the D value of JM418 was the highest, reaching 0.753, followed by SM22, H4399, and HM19 (**Table 6**). SX828 had the lowest D value (0.128). The squared Euclidean distance and the clustering method were used to cluster the average D values for 2 years. At the Euclidean distance of 1.5, 16 wheat varieties could be clustered into 3 categories (**Figure 8**), including drought-resistant (GY2018, HG35, JM418, HM19, SM22, and H4399), drought weak sensitive (GY5218, CM6002, JM585, JM738, S4366, XM7, and SL02–1) and drought-sensitive varieties (SX828, SM13, and SN086).

### 3.6 Screening and evaluation of drought resistance traits

To screen and evaluate drought tolerance traits in winter wheat, we used the D value as a dependent variable and the drought resistance coefficient of each trait as an independent variable to develop the best regression equation for predicting drought tolerance. The optimal regression equation is as follows: D =-4.775+5.056Ci-3.701SN+2.528LWC-1.693POD+4.421PH+0.902MDA-2.76A-2.494SP-0.706ABA+2.37CT (R^2^ = 0.988, P = 0.0001). Grey correlation analysis showed that the correlation coefficient between 24 traits ranged from 0.605 to 0.685, with an average value of 0.653. ABA had the strongest correlation with the comprehensive evaluation value of drought tolerance (D value) (0.685), followed by A (0.669). The traits closely related to the D value were ABA, A, PH, GN, TGW, MDA, Gs, LWC, CT, and Ci. Seven common traits were used in the grey correlation analysis and regression equation, namely ABA, A, PH, MDA, LWC, CT, and Ci, which also confirmed the feasibility and accuracy of the regression equation.

## 4 Discussion

In recent years, climate change has caused a decrease in the yield of gramineous crops, including a 5.5% decrease in the global wheat yield (Lobell et al., 2011). Drought stress has emerged as one of the most significant abiotic stresses limiting crop production globally, and wheat is one of the most vulnerable crops to drought stress (Mei et al., 2022). Many wheat cultivars have been previously cultivated, but the response of different wheat cultivars to drought stress has been strikingly different. To resist climate change and reduce the agricultural water pressure in the NCP, breeding excellent wheat cultivars is an important measure to maintain and even increase wheat yield under adverse weather conditions. Scientists have made significant progress in developing high-yielding wheat cultivars since the Green Revolution. This has, however, been accomplished based on high yields obtained under ideal conditions, with no additional screening for tolerance traits under adverse conditions (Landi et al., 2017). Further progress in the development of drought-resistant germplasm depends on the efficiency of breeding methods and the evaluation of drought tolerance traits, and accurate drought phenotyping implies a precise definition of target environments and the management and characterization of water stress (Monneveux et al., 2012). At present, the evaluated traits associated with wheat drought tolerance are affected by multiple factors, such as experimental environment, screening period, and representativeness of indices. The results are not the same.

In our study, 16 wheat cultivars were cultivated in the field for two consecutive years. Grain yield and 24 traits closely related to drought tolerance, including morphological, physiological, and biochemical traits, were measured. Within 2 years, the results of variance analysis revealed significant yield differences among wheat cultivars grown in different water treatments, and the difference was more obvious under DS. Among them, CM6002, SM22, JM418, and HG35 had the lowest average decrease and showed better drought tolerance. However SX828, SL02–1, and XM7 had the biggest declines and were more sensitive to drought. These indicates that the selected wheat varieties have enough genetic diversity to be regionally representative. Drought stress had a significant effect on 24 traits (P< 0.05), with 18 traits decreasing (drought tolerance coefficient< 1) and 6 traits increasing (drought tolerance coefficient > 1) (**Table 3**). Meanwhile, the coefficient of variation (CV) value of most traits under DS was higher than that under CK, indicating that the wheat cultivars selected in this study were abundant, the effect of drought stress was obvious, and the results were representative.

### 4.1 Evaluation of drought tolerance of wheat by multivariate analysis

To avoid inherent differences among cultivars, the performance of different wheat cultivars under drought stress was evaluated by relative values. However, drought tolerance is a complex trait determined by multiple factors, and several errors occur in evaluating drought tolerance of different cultivars by single-trait or single-type trait. At present, no single trait can be used to fully and accurately evaluate wheat’s drought resistance; therefore, selecting more comprehensive traits and appropriate evaluation methods are very important for the evaluation of wheat cultivars. In addition, there was a certain degree of correlation between many indicators, which led to the overlapping response as a source of crop stress tolerance traits (**Figure 6**). Therefore, it is necessary to use the multivariate analysis method to evaluate and screen for comprehensive traits related to drought resistance. PCA can reduce multiple variables to a few underlying factors while reducing missing data, allowing for more efficient grouping of drought-tolerant genotypes (Wu and Bao, 2012; Maheswari et al., 2016). Through PCA, 24 individual traits of winter wheat under drought stress were converted into 7 independent comprehensive indices (**Table 4**). The cumulative contribution rate of the first 7 independent comprehensive indicators reached more than 80% during the two-year experiment, indicating that most of the data on 24 traits could be covered. The drought tolerance membership function value is a multivariate index that integrates the drought tolerant coefficients of different traits and can effectively reflect the overall performance of plants under drought stress. The membership function values were calculated based on the principal component scores, and the comprehensive evaluation value of drought tolerance (D value) was calculated by combining the weights; the drought tolerance of wheat cultivars was ranked according to the D value. Many previous studies have classified 12 onion cultivars into two groups according to their waterlogging tolerance and wheat and maize salt-tolerant genotypes according to their Euclidean distances (Huqe et al., 2021; Gedam et al., 2022; Uzair et al., 2022). The cotton cultivars were classified according to their drought tolerance by the membership function and D value (Zahid et al., 2021). In this study, wheat cultivars differed significantly in various morphological and physiological characteristics in different growing seasons, indicating that there was sufficient genetic diversity among the selected wheat cultivars. Also, they were representative of the region. We used PCA to convert the 24 drought resistance indicators of wheat into 7 independent composite indicators. D values of different wheat cultivars were obtained by the membership function. Furthermore, the use of PCA in conjunction with the membership function and cluster analysis makes assessing stress resistance in crops more reliable and practical. Hierarchical clustering analysis classified 16 wheat cultivars into 3 categories based on the D value: drought resistant, drought weak sensitive, and drought sensitive. In this study, 10 drought tolerance traits (Ci, SN, LWC, POD, PH, MDA, A, SP, ABA, and CT) were identified by regression analysis and found to have significant effects on drought tolerance of wheat and, therefore, it could be used as the main traits for screening drought tolerant wheat cultivars in the future. At the same time, we established a reliable regression model for the drought resistance evaluation of wheat as follows: D =-4.775+5.056Ci-3.701SN+2.528LWC-1.693POD+4.421PH+0.902MDA-2.76A-2.494SP-0.706ABA+2.37CT (R^2^ = 0.988, P = 0.0001). The grey relational analysis can determine the correlation degree between the drought tolerant coefficient of each trait and the D value. The higher the correlation degree, the stronger the correlation between a trait and drought tolerance of wheat. The results of the grey relational analysis further confirmed the accuracy of the regression analysis and enhanced the scientific reliability and persuasive assessment of the identified traits.

### 4.2 Screening for drought resistance traits under drought stress

Plant growth is an important index to measure drought adaptation, and most scientists evaluate drought-tolerant and drought-sensitive wheat cultivars under drought conditions based on morphological characteristics or yield indicators, including plant height, tiller number, spikelet number, grain number per spike, 1000-grain weight, leaf area index, biological yield, and grain yield (Zhao et al., 2013; Gao et al., 2020; Memon et al., 2021; Ahmad et al., 2022). In this study, we looked at three phenotypic traits: SN, SP, and PH. Drought stress, on the other hand, affects not only plant morphology and yield but also the physiological and biochemical characteristics of plants (Claeys and Inze, 2013). Photosynthesis is an important process for the production of dry matter, and the increase in grain yield is due to the assimilation products of photosynthesis (Li et al., 2020). Water stress during the grain-filling period can lead to decreased photosynthesis, induce accelerated leaf senescence, and shorten the grain-filling period, with the latter being the main reason for the decrease in wheat yield (Yang and Zhang, 2006). During photosynthesis, stomatal traits account for the large extent of yield losses since stomatal closure can reduce water loss (Liu et al., 2010). We identified two important photosynthetic physiological traits, including A and Ci, highly similar to the results of previous studies. Similarly, CT and LWC during the grain-filling period can also be used to identify drought-resistant genotypes. CT is a low-cost, large-scale method for rapidly identifying drought-tolerant cultivars at the canopy level. At the grain-filling stage, there was a continuous negative linear correlation between canopy temperature (CT) and grain yield, and higher leaf water content could contribute to and maintain a lower canopy temperature and a larger water absorption capacity of roots (Deery et al., 2016). Throughout evolution, plants have withstood harsh environmental conditions by elevating abscisic acid levels, controlling stomatal aperture, accumulating antioxidants and osmoprotectants, and regulating gene expression in response to stress (Fu et al., 2016). Therefore, these are often used as physiological indicators associated with stress resistance in crops (Hassan et al., 2015; Mu et al., 2021). Reactive oxygen species (ROS) produced as a result of drought stress lead to lipid peroxidation and increased activities of superoxide dismutase (SOD), peroxidase (POD), catalase (CAT), and ascorbate peroxidase (APX), which are important components of the antioxidant enzyme system in the cell membrane and can reduce the toxicity of ROS to cells. In addition, ABA plays a crucial role in the response of plants to drought through stomatal closure and maintenance of the water balance, as well as the transcription and activity of antioxidant enzymes (Wang et al., 2021). In other words, the improved plant performance provided by these traits can serve as a foundation for selecting materials for the development of drought-tolerant wheat genotypes (Mwadzingeni et al., 2016). Chlorophyll content has traditionally been regarded as a key indicator of wheat growth. The retention of green leaf area was the most valuable genetic trait associated with maintenance of yield under drought conditions (Foulkes et al., 2007). However, SPAD value was not selected as the identification index of drought resistance, which may be due to the difference in wheat leaf color itself, leading to different experimental results. The results of our screening revealed that A, Ci, POD, MDA, and ABA could best reflect drought resistance in wheat. We speculated that flag leaves maintained high anti-aging ability and maintain higher photosynthesis in the late grain filling stage under DS, thus guaranteeing the accumulation of dry matter. This is the key physiological factor to distinguish drought tolerance of different wheat cultivars.

**Figure 9:**
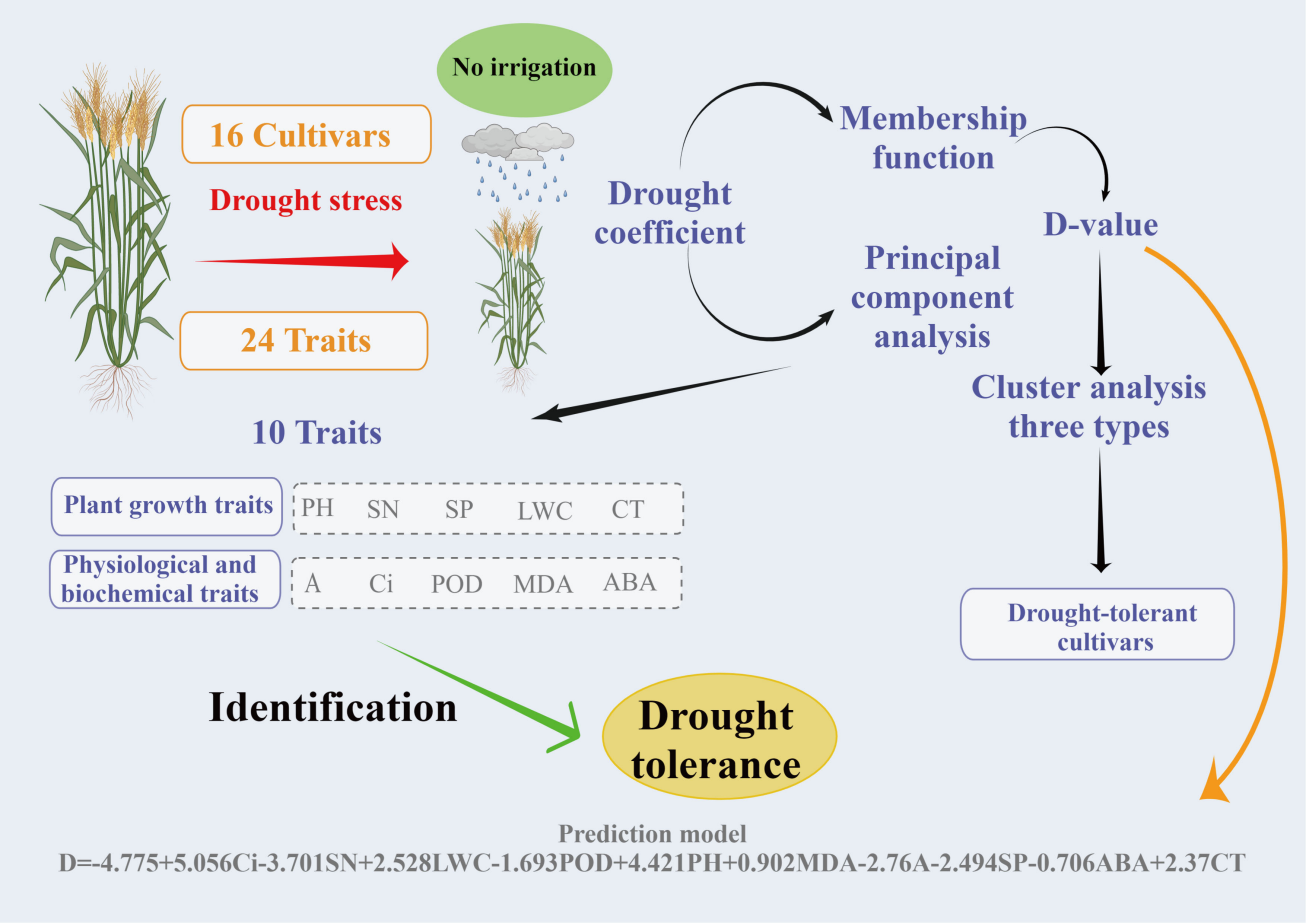
Screening process and prediction model for drought-resistant wheat cultivars and drought-resistance traits.

Through a two-year field experiment, we comprehensively evaluated the drought tolerance of different wheat varieties, identified the wheat varieties with superior drought tolerance, screened 10 key indices for the evaluation of winter wheat drought tolerance, and developed the best mathematical model for the prediction of wheat drought resistance. There are some flaws in this study as well. For example, we only considered variation between indicators for two conditions (normal irrigation and drought stress). However, it has been reported that after being subjected to abiotic stress, some physiological parameters of plants can restore to optimal function(Liu et al., 2022). Therefore, we believe that further research into the resilience of different wheat varieties after drought rehydration compensation and the feasibility of key indicators is required. This could lead to more drought-tolerant varieties being selected. The flow chart of the screening of drought-tolerant cultivars and drought-tolerance traits in **Figure 9** provides a foundation for efficient and accurate identification of drought-tolerant wheat cultivars for future wheat production research.

## 5 Conclusion

This study evaluated 24 drought-related indices, including yield, morphology, photosynthesis, physiology, and osmotic regulation in wheat, under drought stress conditions. PCA, membership function analysis, and multiple regression analysis were performed to effectively evaluate the drought resistance of the wheat crop. A total of 10 traits associated with wheat drought resistance, such as PH, SN, SP, CT, LWC, A, Ci, POD, MDA, and ABA, were evaluated, and a digital model for wheat drought resistance evaluation was established. Furthermore, 6 drought-tolerant wheat cultivars were chosen: JM418, HM19, SM22, HG35, H4399, and CM6002. This study provides useful material for breeding wheat cultivars with drought resistance and the theoretical basis for explaining the mechanism underlying wheat drought resistance.

## Data availability statement

The original contributions presented in the study are included in the article/supplementary material. Further inquiries can be directed to the corresponding authors.

## Author contributions

WZ and LG conceived the project and set scientific objectives. XB, XL, and XH contributed to preparing the field experiment and data acquisition. XB, XL, and XH wrote the manuscript. WD, JR, BY, and YW writing—review and editing. All authors contributed to the article and approved the submitted version.
